# Trajectories of Chronic Disease and Multimorbidity Among Middle-aged and Older Patients at Community Health Centers

**DOI:** 10.1001/jamanetworkopen.2023.7497

**Published:** 2023-04-11

**Authors:** Ana R. Quiñones, Jun Hwang, John Heintzman, Nathalie Huguet, Jennifer A. Lucas, Teresa D. Schmidt, Miguel Marino

**Affiliations:** 1 Department of Family Medicine, Oregon Health & Science University, Portland; 2 OHSU-PSU School of Public Health, Oregon Health & Science University, Portland; 3 Research Department, OCHIN Inc, Portland, Oregon

## Abstract

**Importance:**

Health-related consequences of multimorbidity (≥2 chronic diseases) are well documented. However, the extent and rate of accumulation of chronic diseases among US patients seeking care in safety-net clinics are not well understood. These insights are needed to enable clinicians, administrators, and policy makers to mobilize resources for prevention of disease escalations in this population.

**Objectives:**

To identify the patterns and rate of chronic disease accumulation among middle-aged and older patients seeking care in community health centers, as well as any sociodemographic differences.

**Design, Setting, and Participants:**

This cohort study used electronic health record data from January 1, 2012, to December 31, 2019, on 725 107 adults aged 45 years or older with 2 or more ambulatory care visits in 2 or more distinct years at 657 primary care clinics in the Advancing Data Value Across a National Community Health Center network in 26 US states. Statistical analysis was performed from September 2021 to February 2023.

**Exposures:**

Race and ethnicity, age, insurance coverage, and federal poverty level (FPL).

**Main Outcomes and Measures:**

Patient-level chronic disease burden, operationalized as the sum of 22 chronic diseases recommended by the Multiple Chronic Conditions Framework. Linear mixed models with patient-level random effects adjusted for demographic characteristics and ambulatory visit frequency with time interactions were estimated to compare accrual by race and ethnicity, age, income, and insurance coverage.

**Results:**

The analytic sample included 725 107 patients (417 067 women [57.5%]; 359 255 [49.5%] aged 45-54 years, 242 571 [33.5%] aged 55-64 years, and 123 281 [17.0%] aged ≥65 years). On average, patients started with a mean (SD) of 1.7 (1.7) morbidities and ended with 2.6 (2.0) morbidities over a mean (SD) of 4.2 (2.0) years of follow-up. Compared with non-Hispanic White patients, patients in racial and ethnic minoritized groups had marginally lower adjusted annual rates of accrual of conditions (−0.03 [95% CI, −0.03 to −0.03] for Spanish-preferring Hispanic patients; −0.02 [95% CI, −0.02 to −0.01] for English-preferring Hispanic patients; −0.01 [95% CI, −0.01 to −0.01] for non-Hispanic Black patients; and −0.04 [95% CI, −0.05 to −0.04] for non-Hispanic Asian patients). Older patients accrued conditions at higher annual rates compared with patients 45 to 50 years of age (0.03 [95% CI, 0.02-0.03] for 50-55 years; 0.03 [95% CI, 0.03-0.04] for 55-60 years; 0.04 [95% CI, 0.04-0.04] for 60-65 years; and 0.05 [95% CI, 0.05-0.05] for ≥65 years). Compared with those with higher income (always ≥138% of the FPL), patients with income less than 138% of the FPL (0.04 [95% CI, 0.04-0.05]), mixed income (0.01 [95% CI, 0.01-0.01]), or unknown income levels (0.04 [95% CI, 0.04-0.04]) had higher annual accrual rates. Compared with continuously insured patients, continuously uninsured and discontinuously insured patients had lower annual accrual rates (continuously uninsured, −0.003 [95% CI, –0.005 to –0.001]; discontinuously insured, −0.004 [95% CI, –0.005 to –0.003]).

**Conclusions and Relevance:**

This cohort study of middle-aged patients seeking care in community health centers suggests that disease accrued at high rates for patients’ chronological age. Targeted efforts for chronic disease prevention are needed for patients near or below the poverty line.

## Key Points

**Question:**

Do patients seen in community health centers accumulate chronic diseases early in midlife, and do they accumulate complex patterns of chronic disease?

**Findings:**

In this cohort study of 725 107 patients, those seeking care in safety-net clinics had high and increasing levels of chronic disease and patterns of disease most frequently characterized by cardiometabolic disease (particularly for adults in racial and ethnic minoritized groups) and mental health conditions (particularly for non-Hispanic White adults).

**Meaning:**

This study suggests that patients seeking care in safety-net clinics are accruing chronic diseases at high rates in middle age, indicating a need for earlier and improved chronic disease prevention efforts.

## Teaser

This cohort study uses electronic health record data from 657 primary care clinics to identify the patterns and rate of chronic disease accumulation among middle-aged and older patients seeking care in community health centers, as well as any sociodemographic differences.

## Introduction

Multimorbidity (≥2 chronic diseases)—a major public health concern associated with adverse health outcomes extending beyond risks attributable to any single constituent disease—amounts to substantial costs for patients, health care systems, and public programs.^1,2,3^ The detrimental effects on health outcomes are evident whether multimorbidity involves etiologically concordant or discordant diseases (eg, hypertension and stroke vs hypertension and depression). Multimorbidity is most common among older adults.^4,5^ Approximately 50% of middle-aged adults (50-65 years) and more than 80% of older adults (≥75 years) live with multimorbidity.^3^ These trends highlight the need for adequate health care access in middle to late life (specifically, timely delivery of preventive services to stave off disease onset).

Many of the most socioeconomically vulnerable patients in the US receive care in community health centers (CHCs), federally qualified health centers, and publicly funded community organizations that comprise the US health care safety net, which provides care regardless of health insurance coverage, documentation status, or ability to pay.^6^ These health centers are important care delivery sites for patients with low income who require care for their high burden of chronic diseases.^7,8^ Care in the safety net is characterized by complex biopsychosocial needs deriving from multiple chronic physical diseases, mental health conditions, and social circumstances that require comprehensive services for effective treatment, management, and control.^9,10^ Still, despite advancements in our understanding of how multimorbidity develops, combines, and changes over time,^11,12,13,14,15^ to our knowledge, there has been comparatively little work done to understand how these processes occur for patients in low-income populations receiving care in CHCs.

Studies involving US nationally representative samples of noninstitutionalized adults show that older adults from racial and ethnic minoritized communities are more likely to develop chronic diseases earlier in midlife and live extended periods of their lifespan in sicker and more disabled states.^14,16^ Greater understanding of how multimorbidity develops among adults seeking care from safety-net clinics is necessary to plan for and deliver appropriate and responsive care.^17^ As such, these individuals represent prime targets for programs and interventions aimed at limiting the progression and impact of multimorbidity on more distal outcomes.

This study aims to assess how multimorbidity develops and progresses over time among middle-aged and older patients receiving care at CHCs and whether there are substantial differences in the trajectories of multimorbidity for patients receiving care at CHCs who are from different racial and ethnic backgrounds and socioeconomic status. We used electronic health record (EHR) data to evaluate trajectories of multimorbidity development and progression by these social factors because of the unequal social conditions experienced by patients in racial and ethnic minoritized groups. We hypothesize that patients receiving care at CHCs who are from racial and ethnic minoritized backgrounds, as well as those with lower socioeconomic status, will have greater chronic disease burden and accumulate chronic disease at a higher rate than those from non–racial and ethnic minoritized backgrounds and higher socioeconomic status.

## Methods

### Data Source

The EHR data used in this study are from the Accelerating Data Value Across a National Community Health Center Network (ADVANCE) Clinical Research Network (CRN) in PCORnet,^18^ the National Patient-Centered Clinical Research Network. ADVANCE is led by OCHIN (a collaborative that represents a large CHC network using 1 EHR system) in partnership with Health Choice Network, Fenway Health, and Oregon Health & Science University (OHSU). ADVANCE represents a unique “community laboratory” of clinical data for research with underrepresented populations receiving care in CHCs. The ADVANCE patient population has been found to be representative of the overall US population of patients receiving care at CHCs.^6,19,20^ The study protocol was approved by OHSU’s institutional review board under exemption category 4 (without need to obtain prior consent because the analyses are of secondary data). This study followed the Strengthening the Reporting of Observational Studies in Epidemiology (STROBE) reporting guideline for cohort studies.

### Study Cohort

We assessed data from 725 107 patients in 657 CHC delivery sites providing primary care services throughout the study period from January 1, 2012, to December 31, 2019 (data pulled June 2020), operated by 148 health systems in 26 states: Alaska, California, Connecticut, Florida, Georgia, Hawaii, Indiana, Kansas, Maryland, Massachusetts, Michigan, Minnesota, Missouri, Montana, New Mexico, Nevada, New York, North Carolina, Ohio, Oregon, Rhode Island, South Carolina, Texas, Utah, Washington, and Wisconsin. Patient data were included if patients had 2 or more ambulatory visits across distinct years during the study period, were aged 45 years or older at the first visit date, and had nonmissing race and ethnicity data. We required 2 or more visits across distinct years due to modeling requirements for estimating trajectories of chronic disease. Furthermore, we included adults aged 45 years or older to assess trajectories of chronic disease among a younger middle-aged cohort than is assessed in typical geriatric studies to evaluate chronic disease accumulation that may be occurring at earlier ages for patients receiving care at CHCs. Of the 783 659 patients meeting criteria, 47 812 (6.1%) were missing race and ethnicity information, whereas 10 740 (1.4%) belonged to racial and ethnic groups excluded from analyses due to small sample sizes and lack of interpretable value for this heterogeneous category (eFigure in Supplement 1).

### Dependent Variable

As our primary outcome, we assessed the sum of 22 chronic diseases recommended by the US Department of Health and Human Services (US-DHHS) Multiple Chronic Conditions Framework, adopting the assumption that these conditions have lasting pathophysiological effects, last 1 year or more, require ongoing medical attention, and/or limit activities of daily living^21,22^: anxiety, arthritis, asthma, autism, cancer, cardiac arrhythmia, chronic kidney disease, chronic obstructive pulmonary disease, congestive heart failure, coronary artery disease, dementia, depression, diabetes, hepatitis, HIV, hyperlipidemia, hypertension, osteoporosis, posttraumatic stress disorder, schizophrenia, substance use disorder, and stroke. We ascertained chronic diseases from EHR problem list *International Classification of Diseases, Ninth Revision, Clinical Modification*, and *International Statistical Classification of Diseases and Related Health Problems, Tenth Revision, Clinical Modification*, codes, which we summarized to named conditions, using established Centers for Medicare & Medicaid Services Chronic Condition Warehouse disease algorithms.^23,24^ As problem lists capture only confirmed diagnoses, we did not require multiple codes to assign status. We considered the first occurrence of a code—either the onset date or first reported date if onset date was unavailable—to indicate a condition’s start date. Conditions with no resolve date noted were considered to be ongoing, in accordance with the designed structure of EHR problem list records. Although problem list maintenance can be an issue in EHR systems, we found that problem lists were largely concordant with and meaningfully supplemented other EHR sources for chronic condition ascertainment in our study population.^25^ Once patient conditions were ascertained, the patient was recorded as having the condition from that point forward. Overall counts of chronic diseases for each patient were summarized at the end of each calendar year for the study period.

We present multimorbidity tabulations for the analytic sample by the ends of the first and final years of observation and categorized as mutually exclusive, clinically relevant groups of chronic diseases informed by prior studies of prevalent multimorbidity patterns in this population^17^: (1) no multimorbidity (0 or 1 chronic disease); (2) cardiometabolic multimorbidity (≥2 cardiovascular or metabolic diseases: cardiac arrhythmia, congestive heart failure, coronary artery disease, diabetes, chronic kidney disease, stroke, hypertension, hyperlipidemia, or ≥1 cardiometabolic disease with ≥1 other somatic disease: arthritis, asthma, cancer, chronic obstructive pulmonary disease, hepatitis, HIV, or osteoporosis); (3) other somatic multimorbidity (≥2 other somatic diseases only); (4) mental multimorbidity (≥2 mental or neurodegenerative conditions only: depression, anxiety, posttraumatic stress disorder, substance use disorder, schizophrenia, autism, or dementia); and (5) mental-somatic multimorbidity (≥1 mental and ≥1 cardiometabolic or other somatic conditions). We opted for these groupings to emphasize a parsimonious set of clinically informative, prevalent, and impactful combinations assessed over the study period.

### Independent Variables

We included 3 patient-level variables derived from EHR and billing data. These include self-identified race and ethnicity as recorded in the patient’s medical record and household income and insurance patterns over the study period.

Mutually exclusive indicators were used for non-Hispanic White (White), Spanish-preferring Hispanic, English-preferring Hispanic, non-Hispanic Black (Black), and non-Hispanic Asian (Asian). Household income as percentage of the federal poverty level (FPL) was recorded at each visit, aggregated into annually varying summaries reflecting each calendar year of observation and operationalized with the coverage threshold issued by the US-DHHS for program eligibility: all visits less than 138% of the FPL, intermittently less than 138% of the FPL, all visits 138% or more than the FPL, or never documented. Insurance payer information was documented at each visit (categorized as uninsured or insured), aggregated into annually varying summaries of coverage for each calendar year of observation (continuously uninsured indicated all encounters uninsured; continuously insured indicated all encounters insured by private or public payer; and discontinuously insured indicated a mixture of coverage).

### Covariates

Patient-level covariates were derived from the EHR, including sex, starting year of observation during the study period, age (in years) at the start of observation, fixed effects for the state of most-frequented clinic over the study period, and annually varying summaries of the number of ambulatory care visits for each calendar year of observation, to account for increased opportunities for diagnosis.

### Statistical Analysis

Statistical analysis was performed from September 2021 to February 2023. We summarized patient characteristics overall and by race, ethnicity, and language groups, reporting counts and percentages for categorical variables and mean (SD) values or median values with IQRs for continuous variables. Next, we summarized shifts in multimorbidity categories at the ends of the first and final observed years in the study period using an alluvial plot and accompanying table with counts and percentages quantifying changes between categories.

To evaluate changes in chronic disease over time, we estimated yearly rates of chronic diseases using linear mixed models with patient-level random effects. The specification of a linear mixed model was chosen for appropriateness, ease of interpretation, and computational feasibility given our large data set. Time modeled as a linear term was favored over indicator terms for each year for interpretability of overall trends during the observation period, and model diagnostics indicated that treating time as a linear term was an appropriate functional form. The mean number of chronic diseases accumulated yearly was assessed with time terms in the models: a main effect for time was encoded as the integer value for number of years from initial visit-year until study end, and time-interaction terms for race and ethnicity, age, income, and insurance coverage. We present unconditional (model 0), individually adjusted (models 1a-1d for time interactions with race and ethnicity, age, income, and insurance), and fully adjusted (model 2) models including all independent variables and covariates. Statistical tests were 2-sided. Type I error was set to .05. Analyses were performed using R, version 4.0.1 (R Group for Statistical Computing). A diagram documenting the final study sample is provided in eFigure in Supplement 1. The trajectories of chronic disease accumulation by multimorbidity category are provided in eTable 1 in Supplement 1, sensitivity analyses omitting the number of visits given potential correlation with time and a model testing for sex-time interaction are provided in eTable 2 in Supplement 1, prevalent multimorbidity combinations are provided in eTables 3 and 4 in Supplement 1, patient characteristics by income are provided in eTable 5 in Supplement 1, and patient characteristics by insurance are provided in eTable 6 in Supplement 1.

## Results

**Figure 1.**
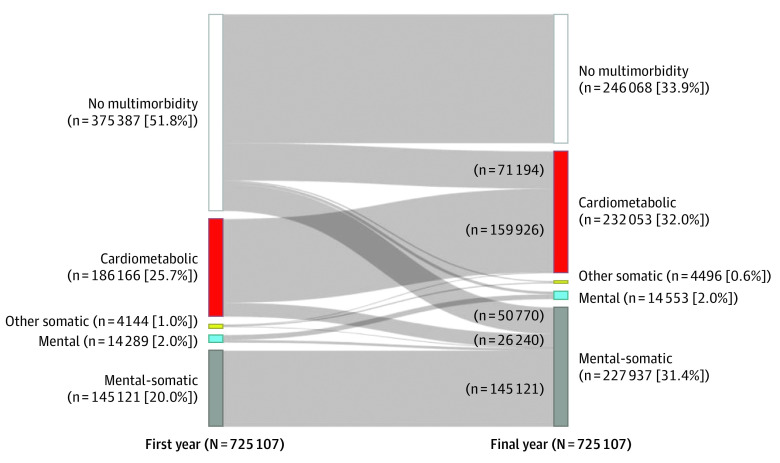
Changes in Patient Multimorbidity Combinations, Accelerating Data Value Across a National Community Health Center Network Clinical Research Network Data, 2012-2019 Patients are grouped in mutually exclusive multimorbidity categories according to chronic disease combinations in the first year of assessment and accounting for accumulation of additional morbidities by the final year of assessment. Because of the chronicity of diseases, patients cannot shift from greater to fewer counts of diseases. Multimorbidity categories are defined as follows: no multimorbidity, 0 or 1 chronic disease; cardiometabolic, 2 or more cardiovascular or metabolic diseases (cardiac arrhythmia, congestive heart failure, coronary artery disease, diabetes, chronic kidney disease, stroke, hypertension, hyperlipidemia, or ≥1 cardiometabolic disease with ≥1 other somatic disease [arthritis, asthma, cancer, chronic obstructive pulmonary disease (COPD), hepatitis, HIV, osteoporosis]); other somatic, 2 or more of arthritis, asthma, cancer, COPD, hepatitis, HIV, or osteoporosis; mental, 2 or more of depression, anxiety, posttraumatic stress disorder, substance use disorder, schizophrenia, autism, and dementia; and mental-somatic, 1 or more mental and 1 or more cardiometabolic or other somatic condition. The remaining numbers of patients flowing from the first to the final year are 933 other somatic to cardiometabolic, 2210 no multimorbidity to other somatic, 2286 other somatic to other somatic, 5145 no multimorbidity to mental, 9408 mental to mental, 925 other somatic to mental-somatic, and 4881 mental to mental-somatic.

Our analytic sample included 725 107 patients with more than 2.7 million observations (417 067 women [57.5%]; 359 255 [49.5%] aged 45-54 years, 242 571 [33.5%] aged 55-64 years, and 123 281 [17.0%] aged ≥65 years) seen in participating CHCs from 2012 to 2019 (Table 1). Overall, patients started with a mean (SD) of 1.7 (1.7) conditions and ended with a mean (SD) of 2.6 (2.0) conditions over a mean (SD) of 4.2 (2.0) years of follow-up over the study period. Figure 1 illustrates change in multimorbidity category for patients seeking care at a CHC in the first and final study years. Although 51.8% of patients (n = 375 387) started with no multimorbidity, 33.9% (n = 246 068) remained without multimorbidity by study end. Of the 375 387 patients who started with no multimorbidity, 65.6% remained in that category, 19.0% shifted to cardiometabolic multimorbidity, and 13.5% shifted to mental-somatic multimorbidity by study end.

**Table 1.** Patient Characteristics by Race and Ethnicity, Accelerating Data Value Across a National Community Health Center Network Clinical Research Network Data, 2012-2019

Characteristic	Overall (N = 725 107)	Non-Hispanic White (n = 314 538)	Spanish-Preferring Hispanic (n = 176 164)	English-Preferring Hispanic (n = 71 731)	Non-Hispanic Black (n = 136 022)	Non-Hispanic Asian (n = 26 652)
Sex, No. (%)						
Female	417 067 (57.5)	174 067 (55.3)	110 344 (62.6)	40 446 (56.4)	76 530 (56.3)	15 680 (58.8)
Male	308 040 (42.5)	140 471 (44.7)	65 820 (37.4)	31 285 (43.6)	59 492 (43.7)	10 972 (41.2)
Baseline age, No. (%), y^a^						
45-54	359 255 (49.5)	142 301 (45.2)	94 014 (53.4)	39 836 (55.5)	72 093 (53.0)	11 011 (41.3)
55-64	242 571 (33.5)	110 649 (35.2)	54 835 (31.1)	22 363 (31.2)	45 674 (33.6)	9050 (34.0)
≥65	123 281 (17.0)	61 588 (19.6)	27 315 (15.5)	9532 (13.3)	18 255 (13.4)	6591 (24.7)
Baseline year FPL, No. (%)^b^						
Continuously ≥138%	432 115 (59.6)	151 830 (48.3)	126 053 (71.6)	46 541 (64.9)	91 890 (67.6)	15 801 (59.3)
Continuously <138%	91 231 (12.6)	50 165 (15.9)	15 866 (9.0)	10 620 (14.8)	12 538 (9.2)	2042 (7.7)
Mixed (over or under 138%)	10 426 (1.4)	5251 (1.7)	2682 (1.5)	859 (1.2)	1301 (1.0)	333 (1.2)
Unknown throughout	191 335 (26.4)	107 292 (34.1)	31 563 (17.9)	13 711 (19.1)	30 293 (22.3)	8476 (31.8)
Baseline year insurance continuity, No. (%)^b^						
Continuously insured	451 293 (62.2)	210 451 (66.9)	95 121 (54.0)	45 692 (63.7)	80 804 (59.4)	19 225 (72.1)
Continuously uninsured	185 996 (25.7)	69 411 (22.1)	56 932 (32.3)	17 726 (24.7)	37 327 (27.4)	4600 (17.3)
Discontinuously insured	87 818 (12.1)	34 676 (11.0)	24 111 (13.7)	8313 (11.6)	17 891 (13.2)	2827 (10.6)
Initial No. of morbidities, mean (SD)^b^						
Total	1.7 (1.7)	1.9 (1.8)	1.5 (1.5)	1.6 (1.6)	1.8 (1.7)	1.7 (1.6)
Cardiometabolic^c^	1.1 (1.2)	1.0 (1.2)	1.1 (1.2)	1.0 (1.2)	1.2 (1.2)	1.2 (1.2)
Other somatic^d^	0.3 (0.6)	0.4 (0.6)	0.2 (0.5)	0.2 (0.5)	0.3 (0.6)	0.3 (0.6)
Mental, psychological, neurologic^e^	0.4 (0.7)	0.5 (0.8)	0.2 (0.5)	0.3 (0.7)	0.3 (0.7)	0.2 (0.6)
Final No. of morbidities, mean (SD)						
Total	2.6 (2.0)	2.7 (2.1)	2.3 (1.8)	2.4 (1.9)	2.6 (1.9)	2.4 (1.8)
Cardiometabolic^c^	1.5 (1.4)	1.4 (1.4)	1.6 (1.3)	1.5 (1.4)	1.7 (1.4)	1.7 (1.4)
Other somatic^d^	0.5 (0.7)	0.6 (0.8)	0.3 (0.6)	0.4 (0.7)	0.5 (0.7)	0.4 (0.7)
Mental, psychological, neurologic^e^	0.6 (0.9)	0.7 (1.0)	0.4 (0.7)	0.5 (0.9)	0.5 (0.9)	0.3 (0.7)
No. of visits, mean (SD)^b^	4.1 (5.4)	4.2 (6.4)	3.9 (3.7)	4.0 (4.4)	4.1 (4.8)	3.9 (6.2)
No. of visits, median (IQR)^b^	3.0 (2.0-5.0)	3.0 (1.0-5.0)	3.0 (2.0-5.0)	3.0 (2.0-5.0)	3.0 (2.0-5.0)	3.0 (2.0-5.0)
Follow-up time, mean (SD), y	4.2 (2.0)	4.2 (2.0)	4.2 (2.0)	4.1 (2.0)	4.2 (2.0)	4.1 (1.9)

Abbreviation: FPL, federal poverty level.

^a^ At first ambulatory visit in the study period.

^b^ During the first year of observation.

^c^ Includes cardiac arrhythmia, congestive heart failure, coronary artery disease, diabetes, chronic kidney disease, stroke, hypertension, and hyperlipidemia.

^d^ Includes arthritis, asthma, cancer, chronic obstructive pulmonary disease, hepatitis, HIV, and osteoporosis.

^e^ Includes depression, anxiety, posttraumatic stress disorder, substance use disorder, schizophrenia, autism, and dementia.

**Figure 2.**
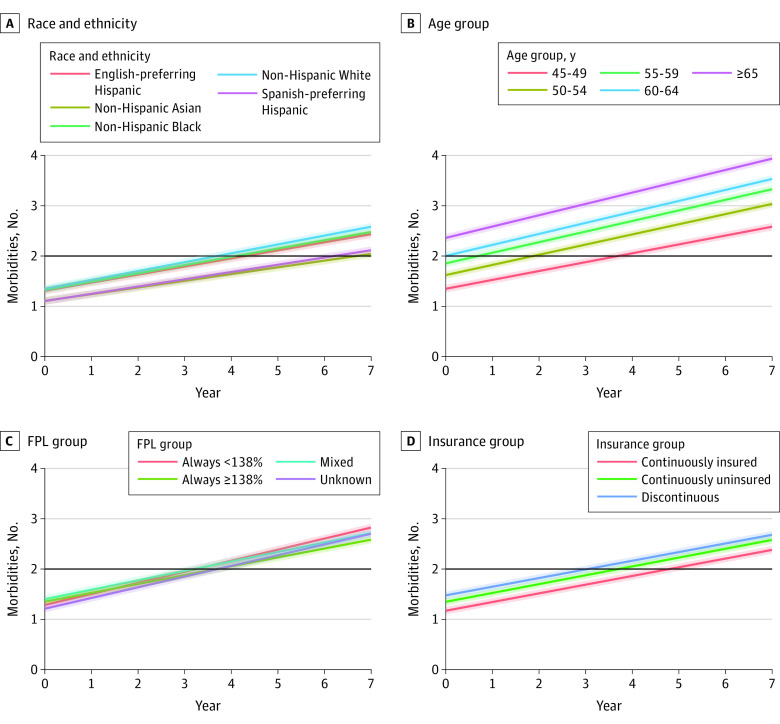
Trajectories of Multimorbidity Accumulation, Accelerating Data Value Across a National Community Health Center Network Clinical Research Network Data, 2012-2019 FPL indicates federal poverty level.

Figure 2 plots the trajectories of chronic disease accumulation by race and ethnicity, age, FPL, and insurance group. With regard to race and ethnicity, Asian and Spanish-preferring Hispanic patients demonstrated the lowest initial levels of chronic disease accumulation and more gradual rates of chronic disease accumulation over the study period. In contrast, White patients demonstrated the highest initial levels of chronic disease accumulation and more accelerated rates of chronic disease increase over the study period, whereas Black and English-preferring Hispanic patients demonstrated trajectories very similar to White patients; these patients crossed the multimorbidity threshold (≥2 chronic diseases), on average, after fewer years of follow-up compared with Asian and Spanish-preferring Hispanic patients (Black patients, 4.1 years; English-preferring Hispanic patients, 4.3 years; White patients, 3.7 years; Asian patients, 6.7 years; and Spanish-preferring Hispanic patients, 6.2 years). An age gradient in the starting level of chronic conditions between older and younger patients was also evident. Although, on average, the oldest age group (≥65 years) already had multimorbidity at the outset of the study period, which increased over time, the youngest age group (45-49 years) crossed the multimorbidity threshold, on average, after a mean of 3.7 years of follow-up. The FPL groups also demonstrated a slight gradient by the end of the follow-up period: those with income less than 138% of the FPL accrued conditions at a higher rate compared with patients whose income was consistently 138% or more of the FPL. Discontinuously insured individuals also demonstrated the highest levels and rates of accumulation of conditions, with continuously insured patients demonstrating the lowest trajectories of chronic disease.

Table 2 presents trajectories of chronic disease accumulation numerically by providing estimated intercepts and slopes for unadjusted and adjusted models. In estimated trajectories of chronic disease accumulation, compared with White patients, those in racial and ethnic minoritized groups had slightly lower adjusted baseline levels (ie, estimated intercepts) of chronic disease. Estimated intercepts were only nominally lower for English-preferring Hispanic patients (*b* = −0.04 [95% CI, −0.05 to −0.02]) and Black patients (*b* = −0.02 [95% CI, −0.03 to −0.01]); however, estimated intercepts were lower for Spanish-preferring Hispanic patients (*b* = −0.24 [95% CI, −0.26 to −0.23]) and Asian patients (*b* = −0.24 [95% CI, −0.26 to −0.22]). Relative to White patients, those in minoritized racial and ethnic groups also demonstrated slower adjusted annual rates of accumulation of conditions, with estimated changes in yearly rates of −0.03 (95% CI, −0.03 to −0.03) for Spanish-preferring Hispanic patients, −0.02 (95% CI, −0.02 to −0.01) for English-preferring Hispanic patients, −0.01 (95% CI, −0.01 to −0.01) for Black patients, and −0.04 (95% CI, −0.05 to −0.04) for Asian patients.

**Table 2.** Linear Mixed Models of Chronic Disease Accumulation Over Time, Accelerating Data Value Across a National Community Health Center Network Clinical Research Network Data, 2012-2019

Covariate	*b* Value (95% CI)					
	Model 0	Model 1a	Model 1b	Model 1c	Model 1d	Model 2^a^
**Baseline count of chronic diseases**						
Intercept	1.76 (1.75 to 1.76)	1.91 (1.90 to 1.91)	1.29 (1.28 to 1.30)	1.83 (1.82 to 1.83)	1.77 (1.77 to 1.78)	1.27 (1.19 to 1.34)
Non-Hispanic White		0 [Reference]				0 [Reference]
Spanish-preferring Hispanic		−0.40 (−0.41 to −0.39)				−0.24 (−0.26 to −0.23)
English-preferring Hispanic		−0.28 (−0.29 to −0.27)				−0.04 (−0.05 to −0.02)
Non-Hispanic Black		−0.09 (−0.10 to −0.08)				−0.02 (−0.03 to −0.01)
Non-Hispanic Asian		−0.19 (−0.21 to −0.17)				−0.24 (−0.26 to −0.22)
Aged ≥45 y and <50 y			0 [Reference]			0 [Reference]
Aged ≥50 y and <55 y			0.33 (0.32 to 0.35)			0.27 (0.26 to 0.28)
Aged ≥55 y and <60 y			0.57 (0.56 to 0.59)			0.51 (0.49 to 0.52)
Aged ≥60 y and <65 y			0.72 (0.71 to 0.74)			0.65 (0.64 to 0.67)
Aged ≥65 y			1.09 (1.08 to 1.11)			1.01 (1.00 to 1.03)
Income always ≥138% of the FPL				0 [Reference]		0 [Reference]
Income always <138% of the FPL				−0.06 (−0.06 to −0.05)		−0.06 (−0.07 to −0.06)
Income mixed FPL				0.09 (0.08 to 0.10)		0.05 (0.04 to 0.06)
FPL unknown throughout				−0.14 (−0.15 to −0.14)		−0.14 (−0.15 to −0.13)
Continuously insured					0 [Reference]	0 [Reference]
Continuously uninsured					−0.17 (−0.18 to −0.17)	−0.18 (−0.18 to −0.17)
Discontinuously insured					0.19 (0.18 to 0.19)	0.13 (0.12 to 0.13)
**Accumulation of chronic diseases**						
Time	0.24 (0.24 to 0.24)	0.25 (0.25 to 0.25)	0.21 (0.21 to 0.22)	0.21 (0.21 to 0.21)	0.23 (0.23 to 0.23)	0.18 (0.17 to 0.18)
Non-Hispanic White		0 [Reference]				0 [Reference]
Spanish-preferring Hispanic × time		−0.03 (−0.03 to −0.03)				−0.03 (−0.03 to −0.03)
English-preferring Hispanic × time		−0.02 (−0.02 to −0.02)				−0.02 (−0.02 to −0.01)
Non-Hispanic Black × time		−0.01 (−0.01 to −0.01)				−0.01 (−0.01 to −0.01)
Non-Hispanic Asian × time		−0.04 (−0.04 to −0.03)				−0.04 (−0.05 to −0.04)
Aged ≥45 y and <55 y			0 [Reference]			0 [Reference]
Aged ≥50 y and <55 y × time			0.03 (0.03 to 0.03)			0.03 (0.02 to 0.03)
Aged ≥55 y and <60 y × time			0.04 (0.04 to 0.04)			0.03 (0.03 to 0.04)
Aged ≥60 y and <65 y × time			0.04 (0.04 to 0.05)			0.04 (0.04 to 0.04)
Aged ≥65 y × time			0.04 (0.04 to 0.05)			0.05 (0.05 to 0.05)
Income always ≥138% of the FPL				0 [Reference]		0 [Reference]
Income always <138% of FPL × time				0.04 (0.04 to 0.04)		0.04 (0.04 to 0.05)
Income mixed FPL × time				0 (0 to 0.01)		0.01 (0.01 to 0.01)
FPL unknown throughout × time				0.03 (0.03 to 0.03)		0.04 (0.04 to 0.04)
Continuously insured					0 [Reference]	0 [Reference]
Continuously uninsured × time					−0.02 (−0.02 to −0.02)	–0.003 (–0.005 to –0.001)
Discontinuously insured × time					−0.02 (−0.02 to −0.02)	–0.004 (–0.005 to –0.003)
**Additional covariates**						
Female						0 [Reference]
Male						0.01 (0 to 0.01)
Number of visits						0.02 (0.02 to 0.02)
Starting year 2012						0 [Reference]
Starting year 2013						−0.46 (−0.47 to −0.44)
Starting year 2014						−0.39 (−0.40 to −0.38)
Starting year 2015						−0.44 (−0.45 to −0.42)
Starting year 2016						−0.34 (−0.36 to −0.33)
Starting year 2017						−0.50 (−0.51 to −0.48)
Starting year 2018						−0.38 (−0.39 to −0.37)

Abbreviation: FPL, federal poverty level.

^a^ Includes state of clinic fixed effects that are not shown; the reference state reflected in the intercept is Florida.

Compared with those aged 45 to 50 years, older patients accumulated conditions at faster annual rates (0.03 [95% CI, 0.02-0.03] for 50-55 years, 0.03 [95% CI, 0.03-0.04] for 55-60 years, 0.04 [95% CI, 0.04-0.04] for 60-65 years, and 0.05 [95% CI, 0.05-0.05] for ≥65 years) (Table 2). Compared with those with income always 138% or more of the FPL, those with income always less than 138% of the FPL (0.04 [95% CI, 0.04-0.05]), mixed income (0.01 [95% CI, 0.01-0.01]), and unknown income levels (0.04 [95% CI, 0.04-0.04]) had higher rates of accumulation of conditions. Compared with continuously insured patients, continuously uninsured and discontinuously insured patients had lower annual rates of accumulation (continuously uninsured, −0.003 [95% CI, –0.005 to –0.001]; discontinuously insured, −0.004 [95% CI, –0.005 to –0.003]). Sensitivity analyses omitting the number of visits and including a time-sex interaction did not substantively change estimates or main findings (eTable 2 in Supplement 1).

## Discussion

This study broadens our understanding of aging among patients seeking care from safety-net clinics by evaluating patterns of multimorbidity and trajectories of chronic disease for middle-aged and older patients seen in CHCs. Although 51.8% of our sample started without multimorbidity, by the end of the study period (4-6 years of follow-up), 66.1% had developed or were living with multimorbidity. These findings highlight a clinically significant increase in multimorbidity burden among predominantly patients with low income who move to a multimorbid state at comparatively young ages—in their mid-40s and early 50s—and accumulate increasingly complex combinations of chronic conditions that are suggestive of older, Medicare-covered populations.^3,14^ We found substantial levels of chronic disease burden and increasing rates of disease accumulation, particularly among younger age groups.^26,27^ We also found that economically vulnerable patients—those below the FPL or with discontinuous insurance coverage—developed multimorbidity at faster rates than more economically stable patients.

Our study aligns with findings that patients seeking care from safety-net clinics are developing chronic disease, multimorbidity, and geriatric conditions prematurely.^7,17,28^ Although longitudinal studies assessing multimorbidity trajectories remain a nascent area in clinical research,^29^ our findings largely align with a study estimating incident multimorbidity that found the risk increased substantially with age and was more likely to develop in midlife.^30^ Our study is also in agreement with others that document increasing prominence of mental health conditions and co-occurrence of mental-somatic multimorbidity treated in CHCs.^9,10,17,28^ In an assessment of UK general practices, Ashworth and colleagues^31^ documented social deprivation patterning of multimorbidity trajectories for adults living in the most socioeconomically deprived areas and among patients from South Asian and Black backgrounds. Both groups were also at highest risk of cardiometabolic multimorbidity.

Our findings documented high and increasing levels of multimorbidity across our sample of patients seeking care at CHCs. The differences observed between English- and Spanish-preferring Hispanic patients are suggestive of acculturative processes that may be protective against large morbidity gains for less-acculturated, Spanish-preferring Hispanic patients. Differences in chronic disease patterns by racial and ethnic group indicated that, while the most prevalent multimorbidity combinations included cardiometabolic and/or mental health conditions for White patients, mental health conditions were much less frequent for Hispanic, Black, and Asian patients (eTable 3 in Supplement 1). Instead, the most prevalent multimorbidity combinations for these groups were dominated by cardiometabolic disease patterns, including kidney system involvement, that were less frequent among White patients.

### Strengths and Limitations

The study has several strengths. First, our study adds to the evolving multimorbidity literature by assessing trends and patterns of chronic disease accumulation for patients reliant on safety-net clinics for their health care. This study contributes to narrowing a substantial gap in our understanding of the changing and dynamic nature of chronic care needs among patients with low income and who are uninsured in the US. By identifying and quantifying patterns and trends in chronic disease accumulation—particularly by salient socioeconomic and demographic characteristics—we parse groups of middle-aged and older patients who experience changes in the rate of accumulation and distinct patterns of concomitant mental and physical conditions to target for future intervention. Second, the large ADVANCE network across multiple years of patient follow-up permits the study of longitudinal changes in the complexity and patterning of multimorbidity for patients with low-income in the US.

This study also has several limitations. First, while there is no standardized way to measure multimorbidity,^32^ we have adopted the framework endorsed by the US-DHHS.^21,22^ Second, our analysis is reliant on patients with multiple clinical encounters to have sufficient numbers of observations to estimate disease accumulation trajectories. Furthermore, the use of diagnoses to measure disease accumulation may not correspond precisely with how and when multimorbidity develops in an individual or population. This criterion may contribute to an underestimation of patterns and trends for patient populations with limited access to clinical services.^33^ In addition, patients may seek care outside of CHCs and be missing or undercounted in analyses. However, several studies demonstrate high patient engagement, continuity, and retention, particularly among patients with chronic diseases.^34,35^ Third, we applied chronic condition algorithms on problem lists for chronic disease ascertainment in our EHR data. Although problem list completeness can be heterogeneous across health care systems and practices, our own studies found that problem lists were highly concordant with many encounter-ascertained diagnoses.^25^ Fourth, our analyses did not assess lifestyle factors, such as smoking or obesity, that may provide insight into the development of several chronic conditions. Future work should focus on disentangling the roles of and interactions between risk factors, impairments, geriatric syndromes (such as frailty), health, and aging in the context of multimorbidity. Similarly, our analyses could not account for access to preventive screenings by sociodemographic groups, which could explain sex, age, and racial and ethnic differences in multimorbidity accumulation. Future work is needed to focus on the mechanisms of chronic disease detection within this population and to identify intervention points.

## Conclusions

US safety-net clinics provide essential care to adults who increasingly contend with multiple health concerns.^8^ Our study found that middle-aged patients seeking care at CHCs have high levels of morbidity for their chronological age. That younger middle-aged cohorts of patients seeking care at CHCs have chronic disease profiles similar to older, Medicare-aged cohorts^14^ speaks to the growing need for chronic and geriatric models of care for patients seeking care from safety-net clinics. Clinics and clinicians have shown inventive and resourceful strategies to care for patients with complex physiological and social needs despite limited resources.^36^ The frequent adoption of care delivery models such as care management, the integration of behavioral health services into primary care, and the connection to wraparound services and community-based programs are critical strategies CHCs will need to use^7,36,37^ to address multimorbidity complexity, particularly for cardiometabolic and mental-somatic combinations of disease evident in patients seeking care at safety-net clinics.

## Notes

Supplement 1. **eFigure.** CONSORT Diagram for Study Analytical Sample **eTable 1.** Linear Mixed Models of Chronic Disease Accumulation Over Time by Multimorbidity Category, ADVANCE Clinical Research Network Data, 2012-2019 **eTable 2.** Linear Mixed Models of Chronic Disease Accumulation Over Time: Comparing Primary Model Results With Models Omitting Number of Ambulatory Visits, and Inclusion of Sex-Time Interaction, ADVANCE Clinical Research Network Data, 2012-2019 **eTable 3.** Top 10 Multimorbidity Combinations by Race/Ethnicity at First and Final Year, ADVANCE Clinical Research Network Data, 2012-2019 **eTable 4.** Top 10 Multimorbidity Combinations by FPL Category at First and Final Year, ADVANCE Clinical Research Network Data, 2012-2019 **eTable 5.** Patient Characteristics by Baseline Year Federal Poverty Level, ADVANCE Clinical Research Network Data, 2012-2019 **eTable 6.** Patient Characteristics by Baseline Year Insurance Continuity, ADVANCE Clinical Research Network Data, 2012-2019

Supplement 2.
Data Sharing Statement
