# Error in End Matter

**DOI:** 10.1001/jamanetworkopen.2023.16304

**Published:** 2023-05-16

In the Original Investigation titled “Trajectories of Chronic Disease and Multimorbidity Among Middle-aged and Older Patients at Community Health Centers,”^1^ published April 11, 2023, information regarding the study’s data use agreement and funding for the data source was inadvertently omitted from the article end matter. This article has been corrected.^1^
