# Supplementary material for: Trajectories of Chronic Disease and Multimorbidity Among Middle-aged and Older Patients at Community Health Centers
Source: JAMA Netw Open. 2023 Apr 11;6(4):e237497. doi: 10.1001/jamanetworkopen.2023.7497 (PMC10091154; doi:10.1001/jamanetworkopen.2023.7497)
Supplement: Supplement 1. — eFigure. CONSORT Diagram for Study Analytical Sample eTable 1. Linear Mixed Models of Chronic Disease Accumulation Over Time by Multimorbidity Category, ADVANCE Clinical Research Network Data, 2012-2019 eTable 2. Linear Mixed Models of Chronic Disease Accumulation Over Time: Comparing Primary Model Results With Models Omitting Number of Ambulatory Visits, and Inclusion of Sex-Time Interaction, ADVANCE Clinical Research Network Data, 2012-2019 eTable 3. Top 10 Multimorbidity Combinations by Race/Ethnicity at First and Final Year, ADVANCE Clinical Research Network Data, 2012-2019 eTable 4. Top 10 Multimorbidity Combinations by FPL Category at First and Final Year, ADVANCE Clinical Research Network Data, 2012-2019 eTable 5. Patient Characteristics by Baseline Year Federal Poverty Level, ADVANCE Clinical Research Network Data, 2012-2019 eTable 6. Patient Characteristics by Baseline Year Insurance Continuity, ADVANCE Clinical Research Network Data, 2012-2019 [file jamanetwopen-e237497-s001.pdf]

Supplementary Online Content

Quiñones AR, Hwang J, Heintzman J, et al. Trajectories of chronic disease and multimorbidity among middle-aged and older patients at community health centers. *JAMA Netw Open.* 2023;6(4):e237497. doi:10.1001/jamanetworkopen.2023.7497

eFigure. CONSORT Diagram for Study Analytical Sample
eTable 1. Linear Mixed Models of Chronic Disease Accumulation Over Time by Multimorbidity Category, ADVANCE Clinical Research Network Data, 2012-2019
eTable 2. Linear Mixed Models of Chronic Disease Accumulation Over Time: Comparing Primary Model Results With Models Omitting Number of Ambulatory Visits, and Inclusion of Sex-Time Interaction, ADVANCE Clinical Research Network Data, 2012-2019
eTable 3. Top 10 Multimorbidity Combinations by Race/Ethnicity at First and Final Year, ADVANCE Clinical Research Network Data, 2012-2019
eTable 4. Top 10 Multimorbidity Combinations by FPL Category at First and Final Year, ADVANCE Clinical Research Network Data, 2012-2019
eTable 5. Patient Characteristics by Baseline Year Federal Poverty Level, ADVANCE Clinical Research Network Data, 2012-2019
eTable 6. Patient Characteristics by Baseline Year Insurance Continuity, ADVANCE Clinical Research Network Data, 2012-2019

This supplementary material has been provided by the authors to give readers additional information about their work.

© 2023 Quiñones AR et al. *JAMA Network Open.*

eFigure. CONSORT Diagram for Study Analytical Sample

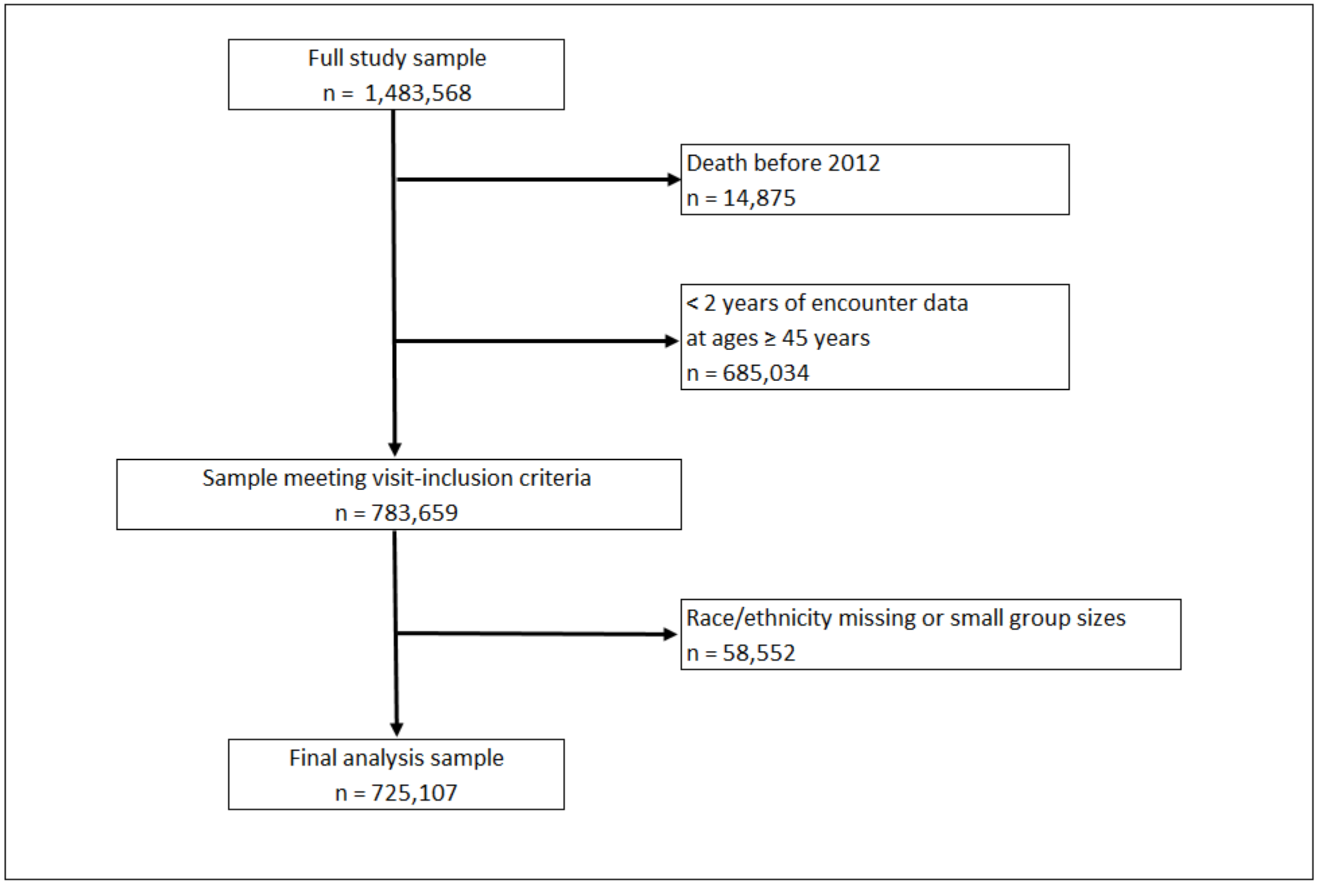

Note: Clinic (delivery site) inclusion criteria were applied to identify the full study sample. We selected clinics that: (1) had primary care, public health, federally qualified health centers (FQHCs), or women's health departments or conducted preventive/wellness visits; (2) were operating on an ADVANCE EHR system during at least a part of the study period; and (3) conducted a 'sufficient volume of care' for at least part of the study period, defined by: (a) serving >50 patients in ambulatory visit (AV) encounters who were ≥45 years of age during the study period (b) offered >50 AV encounters during at least one calendar year in the study period (c) offered >25 clinician (mid-level or above) office visits during at least one calendar year during our study period.

© 2023 Quiñones AR et al. *JAMA Network Open.*

eTable 1. Linear Mixed Models of Chronic Disease Accumulation Over Time by Multimorbidity Category, ADVANCE Clinical Research Network Data, 2012-2019

		**Model for Cardiometabolic**[1]	**Model for Other Somatic**[2]	**Model for Mental**[3]
	**Covariate**	**b (95% CI)**	**b (95% CI)**	**b (95% CI)**
Baseline count of chronic diseases	Intercept	0.64 (0.59, 0.69)	0.26 (0.23, 0.29)	0.37 (0.34, 0.41)
	Non-Hispanic White	Ref	Ref	Ref
	Spanish-preferring Hispanic	0.19 (0.18, 0.19)	-0.16 (-0.17, -0.16)	-0.27 (-0.27, -0.26)
	English-preferring Hispanic	0.13 (0.12, 0.14)	-0.06 (-0.07, -0.06)	-0.11 (-0.11, -0.1)
	Non-Hispanic Black	0.23 (0.22, 0.24)	-0.05 (-0.06, -0.05)	-0.2 (-0.21, -0.2)
	Non-Hispanic Asian	0.22 (0.2, 0.23)	-0.12 (-0.13, -0.12)	-0.33 (-0.34, -0.32)
	Age ≥ 45 and < 50	Ref	Ref	Ref
	Age ≥ 50 and < 55	0.22 (0.21, 0.22)	0.05 (0.05, 0.06)	0 (0, 0.01)
	Age ≥ 55 and < 60	0.42 (0.41, 0.43)	0.1 (0.1, 0.11)	-0.02 (-0.02, -0.01)
	Age ≥ 60 and < 65	0.61 (0.6, 0.62)	0.13 (0.12, 0.13)	-0.08 (-0.09, -0.08)
	Age ≥ 65	0.91 (0.9, 0.92)	0.25 (0.24, 0.25)	-0.14 (-0.15, -0.14)
	FPL always ≥ 138%	Ref	Ref	Ref
	FPL continuously < 138%	-0.02 (-0.03, -0.02)	-0.02 (-0.02, -0.02)	-0.02 (-0.02, -0.02)
	FPL mixed	0.04 (0.04, 0.05)	0 (-0.01, 0)	0.01 (0, 0.01)
	FPL unknown throughout	-0.07 (-0.07, -0.06)	-0.02 (-0.03, -0.02)	-0.05 (-0.05, -0.05)
	Continuously insured	Ref	Ref	Ref
	Continuously uninsured	-0.12 (-0.12, -0.12)	-0.04 (-0.04, -0.04)	-0.02 (-0.03, -0.02)
	Discontinuously insured	0.08 (0.08, 0.09)	0 (0, 0)	0.04 (0.04, 0.04)
Accumulation of chronic diseases	Time	0.08 (0.08, 0.08)	0.04 (0.04, 0.04)	0.06 (0.05, 0.06)
	Non-Hispanic White	Ref	Ref	Ref
	Spanish-preferring Hispanic*Time	0.01 (0.01, 0.01)	-0.02 (-0.02, -0.02)	-0.02 (-0.02, -0.02)
	English-preferring Hispanic*Time	0.01 (0, 0.01)	-0.01 (-0.01, -0.01)	-0.01 (-0.01, -0.01)
	Non-Hispanic Black*Time	0.01 (0.01, 0.01)	-0.01 (-0.01, -0.01)	-0.02 (-0.02, -0.02)
	Non-Hispanic Asian*Time	0.01 (0, 0.01)	-0.02 (-0.02, -0.02)	-0.03 (-0.03, -0.03)

© 2023 Quiñones AR et al. *JAMA Network Open.*

	Covariate	Model for Cardiometabolic[1] b (95% CI)	Model for Other Somatic[2] b (95% CI)	Model for Mental[3] b (95% CI)
	Age ≥ 45 and < 55	Ref	Ref	Ref
	Age ≥ 50 and < 55*Time	0.02 (0.02, 0.02)	0.01 (0.01, 0.01)	0 (0, 0)
	Age ≥ 55 and < 60*Time	0.03 (0.03, 0.03)	0.02 (0.02, 0.02)	-0.01 (-0.01, -0.01)
	Age ≥ 60 and < 65*Time	0.04 (0.03, 0.04)	0.02 (0.02, 0.03)	-0.02 (-0.02, -0.02)
	Age ≥ 65*Time	0.04 (0.04, 0.05)	0.03 (0.03, 0.03)	-0.02 (-0.02, -0.02)
	FPL always ≥ 138%	Ref	Ref	Ref
	FPL continuously < 138%*Time	0.01 (0.01, 0.01)	0.01 (0.01, 0.01)	0.02 (0.02, 0.02)
	FPL mixed*Time	0 (0, 0)	0.01 (0.01, 0.01)	0.01 (0.01, 0.01)
	FPL unknown throughout*Time	0.01 (0.01, 0.02)	0.01 (0.01, 0.01)	0.02 (0.01, 0.02)
	Continuously insured	Ref	Ref	Ref
	Continuously uninsured*Time	0.02 (0.01, 0.02)	-0.01 (-0.01, -0.01)	-0.01 (-0.01, -0.01)
	Discontinuously insured*Time	0 (-0.01, 0)	0 (0, 0)	0 (0, 0)
Additional covariates	Female	Ref	Ref	Ref
	Male	0.18 (0.17, 0.18)	-0.09 (-0.09, -0.08)	-0.08 (-0.09, -0.08)
	Number of visits	0.01 (0.01, 0.01)	0 (0, 0)	0.01 (0.01, 0.01)
	Starting year 2012	Ref	Ref	Ref
	Starting year 2013	-0.26 (-0.27, -0.25)	-0.1 (-0.11, -0.1)	-0.09 (-0.1, -0.09)
	Starting year 2014	-0.23 (-0.24, -0.22)	-0.11 (-0.11, -0.1)	-0.05 (-0.06, -0.05)
	Starting year 2015	-0.26 (-0.27, -0.25)	-0.12 (-0.13, -0.12)	-0.06 (-0.06, -0.05)
	Starting year 2016	-0.26 (-0.26, -0.25)	-0.09 (-0.09, -0.08)	0 (-0.01, 0.01)
	Starting year 2017	-0.35 (-0.36, -0.34)	-0.12 (-0.13, -0.12)	-0.03 (-0.03, -0.02)
	Starting year 2018	-0.26 (-0.27, -0.25)	-0.11 (-0.11, -0.1)	-0.01 (-0.02, -0.01)

Abbreviations: ADVANCE=Accelerating Data Value Across a National Community Health Center Network; FPL=federal poverty level; CI=confidence interval

Notes: Linear mixed models are estimated with patient-level random intercepts; Beta estimates for state of clinic not shown; Reference state reflected in the intercept is Florida

[1]Includes: cardiac arrhythmia, congestive heart failure, coronary artery disease, diabetes, chronic kidney disease, stroke, hypertension, hyperlipidemia

[2]Includes: arthritis, asthma, cancer, chronic obstructive pulmonary disease, hepatitis, HIV, osteoporosis

[3]Includes: depression, anxiety, post-traumatic stress disorder, substance-use disorder, schizophrenia, autism, dementia

© 2023 Quiñones AR et al. *JAMA Network Open.*

eTable 2. Linear Mixed Models of Chronic Disease Accumulation Over Time: Comparing Primary Model Results With Models Omitting Number of Ambulatory Visits, and Inclusion of Sex-Time Interaction, ADVANCE Clinical Research Network Data, 2012-2019

	Covariate	Primary Model Reflected in Manuscript Results<br>b (95% CI)	Sensitivity Analysis Model without Number of Visits<br>b (95% CI)	Sensitivity Analysis Model with Sex*Time Interaction<br>b (95% CI)
Baseline count of chronic diseases	Intercept	1.27 (1.19, 1.34)	1.32 (1.25, 1.4)	1.27 (1.19, 1.34)
	Non-Hispanic White	Ref	Ref	Ref
	Spanish-preferring Hispanic	-0.24 (-0.26, -0.23)	-0.26 (-0.27, -0.25)	-0.24 (-0.25, -0.23)
	English-preferring Hispanic	-0.04 (-0.05, -0.02)	-0.04 (-0.06, -0.03)	-0.04 (-0.05, -0.02)
	Non-Hispanic Black	-0.02 (-0.03, -0.01)	-0.02 (-0.04, -0.01)	-0.02 (-0.03, -0.01)
	Non-Hispanic Asian	-0.24 (-0.26, -0.22)	-0.26 (-0.28, -0.23)	-0.24 (-0.26, -0.22)
	Age ≥ 45 and < 50	Ref	Ref	Ref
	Age ≥ 50 and < 55	0.27 (0.26, 0.28)	0.28 (0.26, 0.29)	0.27 (0.26, 0.28)
	Age ≥ 55 and < 60	0.51 (0.49, 0.52)	0.51 (0.5, 0.52)	0.51 (0.49, 0.52)
	Age ≥ 60 and < 65	0.65 (0.64, 0.67)	0.66 (0.65, 0.67)	0.65 (0.64, 0.67)
	Age ≥ 65	1.01 (1, 1.03)	1.02 (1.01, 1.04)	1.01 (1, 1.03)
	FPL always ≥ 138%	Ref	Ref	Ref
	FPL continuously < 138%	-0.06 (-0.07, -0.06)	-0.06 (-0.06, -0.05)	-0.06 (-0.07, -0.06)
	FPL mixed	0.05 (0.04, 0.06)	0.08 (0.07, 0.09)	0.05 (0.04, 0.06)
	FPL unknown throughout	-0.14 (-0.15, -0.13)	-0.15 (-0.16, -0.14)	-0.14 (-0.15, -0.13)
	Continuously insured	Ref	Ref	Ref
	Continuously uninsured	-0.18 (-0.18, -0.17)	-0.16 (-0.17, -0.16)	-0.18 (-0.18, -0.17)
	Discontinuously insured	0.13 (0.12, 0.13)	0.19 (0.18, 0.19)	0.13 (0.12, 0.13)

© 2023 Quiñones AR et al. *JAMA Network Open.*

	Covariate	Primary Model Reflected in Manuscript Results<br><br>b (95% CI)	Sensitivity Analysis Model without Number of Visits<br><br>b (95% CI)	Sensitivity Analysis Model with Sex*Time Interaction<br><br>b (95% CI)
Accumulation of chronic diseases	Time	0.18 (0.17, 0.18)	0.18 (0.18, 0.18)	0.18 (0.18, 0.18)
	Non-Hispanic White	Ref	Ref	Ref
	Spanish-preferring Hispanic*Time	-0.03 (-0.03, -0.03)	-0.03 (-0.03, -0.03)	-0.03 (-0.03, -0.03)
	English-preferring Hispanic*Time	-0.02 (-0.02, -0.01)	-0.02 (-0.02, -0.01)	-0.02 (-0.02, -0.01)
	Non-Hispanic Black*Time	-0.01 (-0.01, -0.01)	-0.01 (-0.01, -0.01)	-0.01 (-0.01, -0.01)
	Non-Hispanic Asian*Time	-0.04 (-0.05, -0.04)	-0.04 (-0.05, -0.04)	-0.04 (-0.05, -0.04)
	Age ≥ 45 and < 55	Ref	Ref	Ref
	Age ≥ 50 and < 55*Time	0.03 (0.02, 0.03)	0.03 (0.02, 0.03)	0.03 (0.02, 0.03)
	Age ≥ 55 and < 60*Time	0.03 (0.03, 0.04)	0.03 (0.03, 0.04)	0.03 (0.03, 0.04)
	Age ≥ 60 and < 65*Time	0.04 (0.04, 0.04)	0.04 (0.04, 0.04)	0.04 (0.04, 0.04)
	Age ≥ 65*Time	0.05 (0.05, 0.05)	0.05 (0.05, 0.05)	0.05 (0.05, 0.05)
	FPL always ≥ 138%	Ref	Ref	Ref
	FPL continuously < 138%*Time	0.04 (0.04, 0.05)	0.04 (0.04, 0.05)	0.04 (0.04, 0.04)
	FPL mixed*Time	0.01 (0.01, 0.01)	0.01 (0.01, 0.01)	0.01 (0.01, 0.01)
	FPL unknown throughout*Time	0.04 (0.04, 0.04)	0.04 (0.04, 0.04)	0.04 (0.04, 0.04)
	Continuously insured	Ref	Ref	Ref
	Continuously uninsured*Time	0 (0, 0)	0 (-0.01, 0)	0 (0, 0)
	Discontinuously insured*Time	0 (0, 0)	-0.01 (-0.01, -0.01)	0 (-0.01, 0)
Additional covariates	Female	Ref	Ref	Ref
	Male	0.01 (0, 0.01)	0 (0, 0.01)	0.01 (0.01, 0.02)

© 2023 Quiñones AR et al. *JAMA Network Open.*

	Covariate	Primary Model Reflected in Manuscript Results<br>b (95% CI)	Sensitivity Analysis Model without Number of Visits<br>b (95% CI)	Sensitivity Analysis Model with Sex*Time Interaction<br>b (95% CI)
	Male*Time	--	--	0 (0, 0)
	Number of visits	0.02 (0.02, 0.02)	--	0.02 (0.02, 0.02)
	Starting year 2012	Ref	Ref	Ref
	Starting year 2013	-0.46 (-0.47, -0.44)	-0.47 (-0.49, -0.46)	-0.46 (-0.47, -0.44)
	Starting year 2014	-0.39 (-0.4, -0.38)	-0.4 (-0.41, -0.39)	-0.39 (-0.4, -0.38)
	Starting year 2015	-0.44 (-0.45, -0.42)	-0.44 (-0.45, -0.43)	-0.44 (-0.45, -0.42)
	Starting year 2016	-0.34 (-0.36, -0.33)	-0.34 (-0.36, -0.33)	-0.34 (-0.36, -0.33)
	Starting year 2017	-0.5 (-0.51, -0.48)	-0.49 (-0.5, -0.48)	-0.5 (-0.51, -0.48)
	Starting year 2018	-0.38 (-0.39, -0.37)	-0.37 (-0.39, -0.36)	-0.38 (-0.39, -0.37)

Abbreviations: ADVANCE=Accelerating Data Value Across a National Community Health Center Network; FPL=federal poverty level; CI=confidence interval

Notes: Linear mixed models are estimated with patient-level random intercepts; Beta estimates for state of clinic not shown; Reference state reflected in the intercept is Florida

© 2023 Quiñones AR et al. *JAMA Network Open.*

eTable 3. Top 10 Multimorbidity Combinations by Race/Ethnicity at First and Final Year, ADVANCE Clinical Research Network Data, 2012-2019

**Top 10 multimorbidity combinations by the end of the first observation year**	**Overall**	**Non-Hispanic White**	**Spanish-preferring Hispanic**	**English-preferring Hispanic**	**Non-Hispanic Black**	**Non-Hispanic Asian**
	**Rank (%) n=349,720**	**Rank (%) n=161,918**	**Rank (%) n=74,945**	**Rank (%) n=31,884**	**Rank (%) n=68,231**	**Rank (%) n=12,742**
Hypertension, hyperlipidemia	1 (10%)	1 (7.9%)	1 (13.3%)	1 (9.8%)	1 (10.3%)	1 (15.2%)
Diabetes, hypertension, hyperlipidemia	2 (5.6%)	2 (3.1%)	2 (9.1%)	2 (6.2%)	2 (6.7%)	2 (8.8%)
Diabetes, hypertension	3 (3.8%)	4 (1.8%)	3 (5.6%)	3 (4.0%)	3 (6.4%)	3 (4.0%)
Diabetes, hyperlipidemia	4 (2.4%)	8 (1.3%)	4 (5.2%)	4 (2.9%)	9 (1.5%)	4 (3.7%)
Chronic kidney disease, diabetes, hypertension, hyperlipidemia	5 (1.9%)	15 (0.8%)	5 (3.3%)	5 (2.6%)	5 (2.2%)	5 (2.7%)
Hypertension, depression	6 (1.7%)	5 (1.7%)	9 (1.7%)	6 (1.7%)	7 (1.8%)	12 (0.9%)
Anxiety, depression	7 (1.6%)	3 (2.2%)	15 (1.2%)	7 (1.6%)	23.5 (0.6%)	29 (0.5%)
Hypertension, arthritis	8 (1.4%)	9 (1.2%)	12 (1.4%)	12 (1.2%)	4 (2.3%)	13 (0.9%)
Hyperlipidemia, depression	9 (1.3%)	6 (1.5%)	7 (1.7%)	9 (1.4%)	34 (0.4%)	6 (1.4%)
Hypertension, hyperlipidemia, depression	10 (1.3%)	7 (1.5%)	10 (1.6%)	10 (1.4%)	13 (0.8%)	11 (1.0%)
**Top 10 multimorbidity combinations by the end of the last observation year**	**Overall**	**Non-Hispanic White**	**Spanish-preferring Hispanic**	**English-preferring Hispanic**	**Non-Hispanic Black**	**Non-Hispanic Asian**
	**Rank (%) n=479,039**	**Rank (%) n=215,628**	**Rank (%) n=109,035**	**Rank (%) n=44,893**	**Rank (%) n=92,299**	**Rank (%) n=17,184**
Hypertension, hyperlipidemia	1 (7.9%)	1 (6.0%)	1 (10.6%)	1 (7.8%)	1 (8.4%)	1 (12.3%)
Diabetes, hypertension, hyperlipidemia	2 (4.6%)	2 (2.5%)	2 (7.5%)	2 (4.8%)	2 (5.6%)	2 (7.5%)
Chronic kidney disease, diabetes, hypertension, hyperlipidemia	3 (2.5%)	9 (1%)	3 (4.5%)	3 (3.1%)	4 (2.8%)	3 (3.9%)
Diabetes, hypertension	4 (2.2%)	8 (1%)	5 (3.1%)	4 (2.3%)	3 (3.8%)	5 (2.2%)
Diabetes, hyperlipidemia	5 (1.6%)	13 (0.8%)	4 (3.5%)	5 (1.9%)	11 (1.0%)	4 (2.7%)
Hypertension, hyperlipidemia, arthritis	6 (1.4%)	4 (1.3%)	7 (1.7%)	9 (1.2%)	6 (1.5%)	7 (1.2%)

© 2023 Quiñones AR et al. *JAMA Network Open.*

**Top 10 multimorbidity combinations by the end of the last observation year**	**Overall**	**Non-Hispanic White**	**Spanish-preferring Hispanic**	**English-preferring Hispanic**	**Non-Hispanic Black**	**Non-Hispanic Asian**
	**Rank (%) n=479,039**	**Rank (%) n=215,628**	**Rank (%) n=109,035**	**Rank (%) n=44,893**	**Rank (%) n=92,299**	**Rank (%) n=17,184**
Hypertension, hyperlipidemia, depression	7 (1.3%)	5 (1.3%)	8 (1.6%)	6 (1.3%)	13 (0.9%)	13 (0.9%)
Hyperlipidemia, depression	8 (1.2%)	6 (1.2%)	6 (1.7%)	7 (1.3%)	32 (0.4%)	6 (1.2%)
Hypertension, depression	9 (1.1%)	7 (1.1%)	13 (1.2%)	10 (1.1%)	8 (1.3%)	20 (0.7%)
Hypertension, arthritis	10 (1.1%)	10 (0.9%)	15 (1.1%)	12 (0.9%)	5 (1.8%)	17 (0.8%)

© 2023 Quiñones AR et al. *JAMA Network Open*.

eTable 4. Top 10 Multimorbidity Combinations by FPL Category at First and Final Year, ADVANCE Clinical Research Network Data, 2012-2019

Top 10 overall multimorbidity combinations by the end of the first observation year	Overall	Continuously ≥ 138%	Continuously < 138%	Mixed	Undocumented
	Rank (%) n=349,720	Rank (%) n=40,820	Rank (%) n=210,497	Rank (%) n=6,234	Rank (%) n=92,169
Hypertension, hyperlipidemia	1 (10%)	1 (12.2%)	1 (9.6%)	1 (7.7%)	1 (9.9%)
Diabetes, hypertension, hyperlipidemia	2 (5.6%)	2 (5.7%)	2 (5.8%)	2 (5.0%)	2 (5.2%)
Diabetes, hypertension	3 (3.8%)	3 (3.9%)	3 (4.2%)	4 (2.8%)	3 (3.0%)
Diabetes, hyperlipidemia	4 (2.4%)	4 (2.3%)	4 (2.5%)	3 (3%)	4 (2.2%)
Chronic kidney disease, diabetes, hypertension, hyperlipidemia	5 (1.9%)	6 (1.8%)	5 (2.1%)	9 (1.3%)	9 (1.4%)
Hypertension, depression	6 (1.7%)	5 (1.8%)	6 (1.8%)	7 (1.7%)	8 (1.4%)
Anxiety, depression	7 (1.6%)	7 (1.8%)	7 (1.5%)	5 (1.9%)	5 (1.5%)
Hypertension, arthritis	8 (1.4%)	8 (1.6%)	8 (1.4%)	11 (1.1%)	6 (1.5%)
Hyperlipidemia, depression	9 (1.3%)	10 (1.5%)	10 (1.3%)	6 (1.8%)	10 (1.3%)
Hypertension, hyperlipidemia, depression	10 (1.3%)	9 (1.5%)	9 (1.3%)	8 (1.7%)	11 (1.2%)
**Top 10 overall multimorbidity combinations by the end of the last observation year**	**Overall**	**Continuously ≥ 138%**	**Continuously < 138%**	**Mixed**	**Undocumented**
	**Rank (%) n=479,039**	**Rank (%) n=60,491**	**Rank (%) n=282,398**	**Rank (%) n=16,118**	**Rank (%) n=120,032**
Hypertension, hyperlipidemia	1 (7.9%)	1 (9.6%)	1 (7.6%)	1 (6.6%)	1 (8.1%)
Diabetes, hypertension, hyperlipidemia	2 (4.6%)	2 (4.8%)	2 (4.7%)	2 (4.9%)	2 (4.2%)
Chronic kidney disease, diabetes, hypertension, hyperlipidemia	3 (2.5%)	3 (2.2%)	3 (2.7%)	3 (2.6%)	3 (2.1%)
Diabetes, hypertension	4 (2.2%)	4 (2.1%)	4 (2.4%)	5 (2.1%)	4 (1.8%)
Diabetes, hyperlipidemia	5 (1.6%)	5 (1.7%)	5 (1.7%)	4 (2.2%)	6 (1.5%)
Hypertension, hyperlipidemia, arthritis	6 (1.4%)	6 (1.7%)	7 (1.2%)	8 (1.4%)	5 (1.6%)
Hypertension, hyperlipidemia, depression	7 (1.3%)	7 (1.5%)	6 (1.3%)	7 (1.4%)	9.5 (1.1%)
Hyperlipidemia, depression	8 (1.2%)	8 (1.5%)	9 (1.1%)	9 (1.3%)	9.5 (1.1%)
Hypertension, depression	9 (1.1%)	10 (1.2%)	8 (1.2%)	10 (1.1%)	12 (1.0%)
Hypertension, arthritis	10 (1.1%)	11 (1.2%)	10 (1.1%)	16.5 (0.8%)	7 (1.2%)

© 2023 Quiñones AR et al. *JAMA Network Open.*

eTable 5. Patient Characteristics by Baseline Year Federal Poverty Level, ADVANCE Clinical Research Network Data, 2012-2019

	**Overall** n = 725,107	**Continuously ≥138%** n = 91,231	**Continuously <138%** n = 432,115	**Mixed (Over/Under 138%)** n = 10,426	**Unknown throughout** n = 191,335
Race, n (%)					
Non-Hispanic White	314,538 (43.4%)	50,165 (55.0%)	151,830 (35.1%)	5,251 (50.4%)	107,292 (56.1%)
Spanish-Preferring Hispanic	176,164 (24.3%)	15,866 (17.4%)	126,053 (29.2%)	2,682 (25.7%)	31,563 (16.5%)
English-Preferring Hispanic	71,731 (9.9%)	10,620 (11.6%)	46,541 (10.8%)	859 (8.2%)	13,711 (7.2%)
Non-Hispanic Black	136,022 (18.8%)	12,538 (13.7%)	91,890 (21.3%)	1,301 (12.5%)	30,293 (15.8%)
Non-Hispanic Asian	26,652 (3.7%)	2,042 (2.2%)	15,801 (3.7%)	333 (3.2%)	8,476 (4.4%)
Female, n (%)	417,067 (57.5%)	50,152 (55.0%)	252,128 (58.3%)	6,027 (57.8%)	108,760 (56.8%)
Baseline Age[1], n (%)					
45 - 54	359,255 (49.5%)	41,897 (45.9%)	230,210 (53.3%)	6,252 (60.0%)	80,896 (42.3%)
55 - 64	242,571 (33.5%)	32,104 (35.2%)	144,566 (33.5%)	3,138 (30.1%)	62,763 (32.8%)
65 and older	123,281 (17.0%)	17,230 (18.9%)	57,339 (13.3%)	1,036 (9.9%)	47,676 (24.9%)
Baseline year insurance continuity[2], n (%)					
Continuously insured	451,293 (62.2%)	61,929 (67.9%)	237,562 (55.0%)	4,950 (47.5%)	146,852 (76.8%)
Continuously uninsured	185,996 (25.7%)	20,212 (22.2%)	132,409 (30.6%)	2,795 (26.8%)	30,580 (16.0%)
Discontinuously insured	87,818 (12.1%)	9,090 (10.0%)	62,144 (14.4%)	2,681 (25.7%)	13,903 (7.3%)
Initial number of morbidities[2], mean (SD)					
Total	1.7 (1.7)	1.6 (1.6)	1.8 (1.7)	2.2 (1.7)	1.8 (1.7)
Cardiometabolic	1.1 (1.2)	1.0 (1.2)	1.1 (1.2)	1.2 (1.2)	1.1 (1.2)
Other somatic	0.3 (0.6)	0.3 (0.5)	0.3 (0.6)	0.4 (0.6)	0.3 (0.6)
Mental, psychological, neurological	0.4 (0.7)	0.3 (0.6)	0.4 (0.7)	0.6 (0.9)	0.4 (0.7)
Final number of morbidities, mean (SD)					
Total	2.6 (2.0)	2.3 (1.9)	2.6 (2.0)	2.9 (2.0)	2.6 (2.0)
Cardiometabolic[3]	1.5 (1.4)	1.4 (1.3)	1.5 (1.4)	1.6 (1.4)	1.5 (1.4)
Other somatic[4]	0.5 (0.7)	0.4 (0.7)	0.5 (0.7)	0.5 (0.8)	0.5 (0.7)

© 2023 Quiñones AR et al. *JAMA Network Open.*

	Overall<br><br>n = 725,107	Continuously ≥138%<br><br>n = 91,231	Continuously <138%<br><br>n = 432,115	Mixed (Over/Under 138%)<br><br>n = 10,426	Unknown throughout<br><br>n = 191,335
Final number of morbidities, mean (SD)					
Mental, psychological, neurological[5]	0.6 (0.9)	0.4 (0.8)	0.6 (0.9)	0.8 (1.0)	0.6 (0.9)
Number of visits[2], mean (SD)	4.1 (5.4)	3.5 (3.8)	4.3 (6.0)	6.5 (7.0)	3.7 (4.3)
Number of visits[2], median (IQR)	3.0 (2.0, 5.0)	3.0 (1.0, 4.0)	3.0 (2.0, 5.0)	5.0 (3.0, 8.0)	3.0 (1.0, 5.0)
Follow-up time in years, mean (SD)	4.2 (2.0)	4.3 (2.1)	4.2 (2.0)	4.2 (2.1)	4.1 (1.9)

Abbreviations: ADVANCE= Accelerating Data Value Across a National Community Health Center Network; FPL=federal poverty level; SD=standard deviation; IQR=interquartile range

[1] At first ambulatory visit in the study period

[2] During the first year of observation

[3] Includes: cardiac arrhythmia, congestive heart failure, coronary artery disease, diabetes, chronic kidney disease, stroke, hypertension, hyperlipidemia

[4] Includes: arthritis, asthma, cancer, chronic obstructive pulmonary disease, hepatitis, HIV, osteoporosis

[5] Includes: depression, anxiety, post-traumatic stress disorder, substance-use disorder, schizophrenia, autism, dementia

© 2023 Quiñones AR et al. *JAMA Network Open.*

eTable 6. Patient Characteristics by Baseline Year Insurance Continuity, ADVANCE Clinical Research Network Data, 2012-2019

	**Overall**<br>**n = 725,107**	**Continuously insured**<br>**n = 451,293**	**Continuously uninsured**<br>**n = 185,996**	**Discontinuously insured**<br>**n = 87,818**
Race, n (%)				
Non-Hispanic White	314,538 (43.4%)	210,451 (46.6%)	69,411 (37.3%)	34,676 (39.5%)
Spanish-Preferring Hispanic	176,164 (24.3%)	95,121 (21.1%)	56,932 (30.6%)	24,111 (27.5%)
English-Preferring Hispanic	71,731 (9.9%)	45,692 (10.1%)	17,726 (9.5%)	8,313 (9.5%)
Non-Hispanic Black	136,022 (18.8%)	80,804 (17.9%)	37,327 (20.1%)	17,891 (20.4%)
Non-Hispanic Asian	26,652 (3.7%)	19,225 (4.3%)	4,600 (2.5%)	2,827 (3.2%)
Female, n (%)	417,067 (57.5%)	255,195 (56.5%)	107,506 (57.8%)	54,366 (61.9%)
Baseline Age[1], n (%)				
45 - 54	359,255 (49.5%)	205,932 (45.6%)	105,385 (56.7%)	47,938 (54.6%)
55 - 64	242,571 (33.5%)	149,816 (33.2%)	63,373 (34.1%)	29,382 (33.5%)
65 and older	123,281 (17.0%)	95,545 (21.2%)	17,238 (9.3%)	10,498 (12.0%)
Baseline year FPL[2], n (%)				
Continuously ≥138%	91,231 (12.6%)	61,929 (13.7%)	20,212 (10.9%)	9,090 (10.4%)
Continuously <138%	432,115 (59.6%)	237,562 (52.6%)	132,409 (71.2%)	62,144 (70.8%)
Mixed (Over/Under 138%)	10,426 (1.4%)	4,950 (1.1%)	2,795 (1.5%)	2,681 (3.1%)
Unknown throughout	191,335 (26.4%)	146,852 (32.5%)	30,580 (16.4%)	13,903 (15.8%)
Initial number of morbidities[2], mean (SD)				
Total	1.7 (1.7)	1.8 (1.7)	1.3 (1.4)	2.1 (1.7)
Cardiometabolic	1.1 (1.2)	1.1 (1.2)	0.9 (1.1)	1.2 (1.2)
Other somatic	0.3 (0.6)	0.3 (0.6)	0.2 (0.4)	0.3 (0.6)
Mental, psychological, neurological	0.4 (0.7)	0.4 (0.7)	0.3 (0.6)	0.5 (0.9)
Final number of morbidities, mean (SD)				
Total	2.6 (2.0)	2.6 (2.0)	2.2 (1.8)	2.9 (2.0)
Cardiometabolic[3]	1.5 (1.4)	1.5 (1.4)	1.4 (1.3)	1.6 (1.4)
Other somatic[4]	0.5 (0.7)	0.5 (0.8)	0.3 (0.6)	0.5 (0.8)

© 2023 Quiñones AR et al. *JAMA Network Open.*

	Overall<br><br>n = 725,107	Continuously insured<br>n = 451,293	Continuously uninsured<br>n = 185,996	Discontinuously insured<br>n = 87,818
Final number of morbidities, mean (SD)				
Mental, psychological, neurological[5]	0.6 (0.9)	0.6 (0.9)	0.4 (0.8)	0.7 (1.0)
Number of visits[2], mean (SD)	4.1 (5.4)	3.8 (3.6)	3.2 (3.6)	7.4 (11.4)
Number of visits[2], median (IQR)	3.0 (2.0, 5.0)	3.0 (1.0, 5.0)	2.0 (1.0, 4.0)	5.0 (3.0, 8.0)
Follow-up time in years, mean (SD)	4.2 (2.0)	4.1 (2.0)	4.3 (2.1)	4.1 (2.0)

Abbreviations: ADVANCE= Accelerating Data Value Across a National Community Health Center Network; FPL=federal poverty level; SD=standard deviation; IQR=interquartile range

[1] At first ambulatory visit in the study period

[2] During the first year of observation

[3] Includes: cardiac arrhythmia, congestive heart failure, coronary artery disease, diabetes, chronic kidney disease, stroke, hypertension, hyperlipidemia

[4] Includes: arthritis, asthma, cancer, chronic obstructive pulmonary disease, hepatitis, HIV, osteoporosis

[5] Includes: depression, anxiety, post-traumatic stress disorder, substance-use disorder, schizophrenia, autism, dementia

© 2023 Quiñones AR et al. *JAMA Network Open.*
