# Supplementary material for: Trajectories of Chronic Disease and Multimorbidity Among Middle-aged and Older Patients at Community Health Centers
Source: JAMA Netw Open. 2023 Apr 11;6(4):e237497. doi: 10.1001/jamanetworkopen.2023.7497 (PMC10091154; doi:10.1001/jamanetworkopen.2023.7497)
Supplement: Supplement 2. — Data Sharing Statement [file jamanetwopen-e237497-s002.pdf]

Data Sharing Statement

Quiñones. Trajectories of Chronic Disease and Multimorbidity Among Middle-aged and Older Patients at Community Health Centers. *JAMA Netw Open*. Published April 11, 2023. doi:10.1001/jamanetworkopen.2023.7497

Data
Data available: No

Additional Information
Explanation for why data not available: Raw data underlying this article were generated from multiple health systems across institutions in the ADVANCE network; restrictions apply to the availability and re-release of data under organizational data use agreements.
